# Transcutaneous carbon dioxide monitoring: ready to be standard of care?

**DOI:** 10.1038/s41390-026-04910-y

**Published:** 2026-03-15

**Authors:** Annemarie van de Geer, Martijn Miedema, David G. Tingay

**Affiliations:** 1 Department of Neonatology, Amsterdam UMC, University of Amsterdam, Amsterdam, The Netherlands; 2 Amsterdam Reproductive and Development Research Institute, Amsterdam, The Netherlands; 3 Neonatal Research, Murdoch Children’s Research Institute, Parkville, VIC, Australia; 4 Department of Paediatrics, University of Melbourne, Melbourne, VIC, Australia; 5 Department of Critical Care, University of Melbourne, Melbourne, VIC, Australia

Tight control of respiratory gases is a tenant of good critical care, particularly in very low birth weight (VLBW) infants. In these infants, high and low oxygen (O_2_) and carbon dioxide (CO_2_) levels are associated with increased mortality and lung injury as well as preterm specific risks such as intraventricular haemorrhage (IVH), chronic lung disease and retinopathy of prematurity (ROP).^1,2^ Achieving tight O_2_ and CO_2_ control requires knowing what values to target and having a method to monitor levels. Blood gas analysis remains the gold standard method of assessing O_2_ and CO_2_ but does not allow the frequency needed to address the dynamic nature of critical care, leave clinicians effectively blind to rapid fluctuations. Continuous monitoring of O_2_ with peripheral oxygen saturation (SpO_2_) has been standard of care in NICU for more than 20 years. Achieving the same with CO_2_ has been less widely adopted despite the availability of end-tidal and transcutaneous CO_2_ (TcCO_2_) systems.^3^

HFOV is often employed as a rescue strategy in case of severe respiratory failure. HFOV provides efficient alveolar gas exchange by delivering very small tidal volumes at rapid frequencies, theoretically minimising volutrauma and barotrauma.^4^ Due to its potent CO_2_ clearing capacities, HFOV potentially carries greater risk of rapidly fluctuating arterial CO₂ levels. In this edition of *Pediatric Research*, Bernatzky and colleagues report their experiences changing practice to include TcCO_2_ as standard of care for very low birth weight (VLBW) infants receiving high-frequency oscillatory (HFOV).^5^ They compared two populations managed in their NICU (Buenos Aires, Argentina) before (n = 28; 2011-2014) and after (n = 50; 2015-2022) instigating TcCO_2_ monitoring. HFOV was applied universally as a rescue strategy and with an open lung strategy at initiation. All infants received HFOV via the same oscillator (SLE5000, SLE UK Ltd, Croydon, United Kingdom) and TcCO_2_ monitor (Sentec AG, Therwill, Switzerland). Hypocarbia was defined as <40 mmHg and hypercarbia as >55 or >60 mmHg depending on the pH at the time. The primary outcome was the number of hypo- or hypercarbia events per HFOV day. Secondary outcomes included number of blood gas analyses per HFOV day and rates of common complications of critical illness and respiratory support; IVH, mortality, number of transfusions, bronchopulmonary dysplasia, ROP and necrotising enterocolitis.

The two cohorts were reasonably well matched. The introduction of TcCO_2_ monitoring during HFOV was associated with significantly fewer episodes of hypo- or hypercarbia episodes per HFOV; median (IQR) 0.2 (0-0.5) vs 1 (0.4-2) for hypocarbia and 0.5 (0.3-1) vs 1.6 (0.9-2.6) for hypercarbia. The number of blood gas samples was also reduced from 5 (3.5-7) to 3 (2.4-4) samples/HFOV day. Except for hypocarbia episodes this difference was greater in infants born ≤26 weeks’ gestation or ≤1000 g. Approximately 60% of infants in both groups required inotropic support, suggesting that TcCO_2_ monitoring remained informative even in the presence of compromised peripheral perfusion. There was no difference in the rates of all other secondary outcomes except a statistical reduction in the rates of any IVH (but not IVH grade ≥3); mean (SD) 18 (64)% vs 17 (34)% for pre- and TcCO_2_ cohorts.

The authors conclude that TcCO_2_ monitoring during HFOV allowed for more precise management of oscillator settings, which lead to more stable CO_2_ levels. This is consistent with previous studies of TcCO_2_ and asks the question; why is use not more universally accepted?^3,6^ The answer is likely multifactorial. Transcutaneous gas technology is significantly more complicated than SpO_2_ and application requires more operator skill.^3,7,8^ TcCO_2_ sensors need to be calibrated and adjusted every few hours. This led to concerns regarding potential inaccuracy.^9^ TcCO_2_ also requires skin heating to be accurate, old systems were associated with skin complications in the most preterm infants, who are at greatest risk of complications of abnormal CO_2_ levels. All these issues are significantly reduced in current TcCO_2_ systems but it has almost certainty led to a legacy impact.^10^ The availability of volume targeted ventilation (VTV) has also reduced the need to monitor CO_2_ constantly. VTV modes of conventional ventilation are associated with less CO_2_ instability and reduced rates of IVH, BPD and air leak. Until recently, HFOV could not be applied with VTV modes, and use is still limited. HFOV can cause large and rapid changes in CO_2_ compared to conventional and non-invasive ventilation.^11,12^ As such there is greater need for TcCO_2_ during HFOV where mean airway pressure, frequency and amplitude all exert a large impact on CO_2_ levels.^4,11,12^

CO_2_ stability is a sensible primary outcome for a study reporting the use of a monitoring technology in a retrospective cohort study. TcCO_2_ is not an intervention but a monitoring tool that requires clinicians to interpret and then determine an appropriate response, both in terms of the safe CO_2_ range and how to respond if inside or outside that range. The secondary outcome of number of blood gas samples per HFOV is also important. Blood gas sampling comes with complications and cost. If continuous CO_2_ monitoring did not reduce the number of blood samples, it suggests limited utility and lack of trust amongst the clinical staff.^7,8^ It also provides a measure of training, competency and understanding of a new technology amongst clinicians, all of which are essential for successful adoption and translation into practice.

As a single centre retrospective cohort study conducted in a relatively small population over a long period of time, the interpretation of Bernatzky and colleagues study needs to acknowledge the high risk of biases. To understand whether TcCO_2_ was the factor in more stable CO_2_ levels and reduced blood samples, or just an association, requires considering changes in NICU practice, staff levels, skills and training and other monitoring and/or therapies introduced during the same periods. It would be encouraging to conclude that TcCO_2_ reduced the rates of IVH. As this finding is biologically plausible, given the well-established link between CO₂ extremes, CO₂ fluctuations, and IVH risk,^8^ causality cannot be inferred from this small, observational study. A difference in IVH rate of 1% between two cohorts with large standard deviations (that were also a median 1 week different in gestational age) is unlikely to be clinically relevant. Rather these results serve as an illustration of clinical versus statistical significance in observational studies.

There is a strong argument for TcCO_2_ being standard of care in high-risk infants, just as SpO_2_ is. This study adds further weight to this argument. But monitoring CO_2_ is not the same as knowing what CO_2_ to target. This is the more challenging question for clinicians. Permissive hypercapnia has long been the practice, and with good reason.^13^ Permissive hypercapnia acts as a proxy for lower ventilator settings and thus quicker extubation without increasing mortality or chronic lung disease, although the trial data is limited.^13^ Now that TcCO_2_ monitoring is effective and accurate the opportunity for clinicians and researchers to re-evaluate what CO_2_ levels are best exists.
